# An international comparative study of blood pressure in populations of European vs. African descent

**DOI:** 10.1186/1741-7015-3-2

**Published:** 2005-01-05

**Authors:** Richard S Cooper, Katharina Wolf-Maier, Amy Luke, Adebowale Adeyemo, José R Banegas, Terrence Forrester, Simona Giampaoli, Michel Joffres, Mika Kastarinen, Paola Primatesta, Birgitta Stegmayr, Michael Thamm

**Affiliations:** 1 Department of Preventive Medicine and Epidemiology, Loyola University Stritch School of Medicine, Maywood, IL, USA; 2 Department of Pediatrics, University College Hospital, Ibadan, Nigeria; 3 Departamento de Medicina Preventiva y Salud Pública, Facultad de Medicina. Universidad Autónoma de Madrid, Spain; 4 Tropical Medicine Research Institute, University of the West Indies, Kingston, Jamaica; 5 Istituto Superiore di Sanità, Laboratorio di Epidemiologia e Biostatistica, Rome, Italy; 6 Department of Community Health and Epidemiology, Faculty of Medicine, Dalhousie University, Halifax, Nova Scotia, Canada; 7 Department of Public Health and General Practice, University of Kuopio, Finland; 8 Department of Epidemiology and Public Health, University College London Medical School, London, UK; 9 Department of Medicine, University Hospital, Umeå, Sweden; 10 Robert-Koch Institut, Berlin, Germany

## Abstract

**Background:**

The consistent finding of higher prevalence of hypertension in US blacks compared to whites has led to speculation that African-origin populations are particularly susceptible to this condition. Large surveys now provide new information on this issue.

**Methods:**

Using a standardized analysis strategy we examined prevalence estimates for 8 white and 3 black populations (N = 85,000 participants).

**Results:**

The range in hypertension prevalence was from 27 to 55% for whites and 14 to 44% for blacks.

**Conclusions:**

These data demonstrate that not only is there a wide variation in hypertension prevalence among both racial groups, the rates among blacks are not unusually high when viewed internationally. These data suggest that the impact of environmental factors among both populations may have been under-appreciated.

## Background

Population surveys in the US from early in the last century have consistently documented higher blood pressures and related cardiovascular sequelae in blacks compared to whites [1,2]. The enormous attention focused on this observation has resulted in a dichotomous view of hypertension risk: whereby populations of African origin are considered more susceptible than all other continental groupings and a strong genetic hypothesis of inherent predisposition to hypertension among blacks has become the conventional wisdom [3-5]. Since this research has been limited primarily to the US, the generalizability of these conclusions is open to question. Data on the prevalence of hypertension in other genetically-related populations of African and European descent constitute important evidence but have so far not been considered in the debate.

International comparative studies on hypertension have been seriously limited by the absence of a valid method of standardization. In the last decade, however, high quality population surveys have been conducted in a wide range of populations that used either careful internal standardization or sufficiently comparable methods [6-15]. We report here on the patterns of hypertension prevalence in a sample of 3 such surveys among blacks from Africa, the Caribbean and the US and 8 surveys among whites from the US, Canada and Europe.

## Methods

### Study design

Black populations were drawn from the International Collaborative Study on Hypertension (ICSHIB) and the National Health and Nutrition Survey III [6,16]. A primary report of ICSHIB demonstrated a gradient in hypertension risk from east to west, parallel to the gradient in socioeconomic development and associated lifestyle [6]. An extensive process of cross-standardization was incorporated into ICSHIB to ensure that measurement technique did not bias the survey results [7]. We subsequently identified surveys on hypertension conducted since 1986 that were national in scope in North America and Europe. Two North American and six European surveys were included, viz: US [8] and Canada, [9], England [10], Finland [11], Germany [12], Italy [13], Spain [14] and Sweden [15]. The US data from NHANES-III are available for public use through the National Center for Health Statistics [8]. Investigators in Canada and Europe were contacted and invited to join this project. More detailed methods for this component of the study were reported earlier [16]. In brief, after achieving consensus on the main goals and resolving the methodological issues, data collection forms were distributed. Each collaborator provided average gender- and age-specific data by 5-year age groups for BPs, body mass index (BMI), and counts of hypertensives by treatment and control status. A description of the key aspects of each survey, including the BP measurement procedure, was collected in a standardized format.

The surveys that formed the basis of ICSHIB were conducted in localized communities by door-to-door screening [6,7]. In summary, individual communities were chosen on the basis of apparent representativeness and census data were obtained. Sampling was based on probability proportional to size and was structured to lead to a sample equally balanced by gender and age group across the 10-year age. The studies of the European-origin populations and African Americans were larger in scope [16]. Some were based on a random probability sample of the entire nation, while others were a series of regional samples; none were restricted mainly to a single province or sub-region within the country (Table 3). Collectively the studies enrolled 85,000 participants and the number of subjects in individual studies ranged from 1,800 to 23,000. Participation rates varied from 61% to 88%. Sampling was conducted mainly on population registries.

**Table 3:** Hypertension Prevalence (%) among Persons 35–64 Years, in African- and European-Origin Populations *

	**Total (%)**	**Men (%)**	**Women (%)**
**African-Origin Populations**			
Nigeria	13.5	13.9	13.1
Jamaica	28.6	23.4	31.8
US – Black	44.0	43.1	44.8
**European-Origin Populations**			
US – White	26.8	29.7	23.9
Canada	27.4	31.0	23.8
Italy	41.5	48.0	35.1
Sweden	38.4	44.8	32.0
England	41.7	46.9	36.5
Spain	46.8	49.0	44.6
Finland	48.6	55.7	41.6
Germany	55.3	60.2	50.4

* Age-adjusted

### Data collection methods

The examination methods have been reported in detail previously [6,7,16]. In brief, the mercury sphygmomanometer was used for BP measurements in every country except England, where the Dinamap 8100 oscillometric device was used. All studies had at least 2 measurements and the 2^nd ^BP from the clinic visit was used to create the mean for the age-gender groups, except for England where the 2^nd ^home BP was used. Hypertension was defined as BP ≥ 140/90 mmHg or current use of antihypertensive medication.

### Data analysis

BP, body mass index (BMI), and hypertension prevalence were calculated for 5-year age-gender groups and aggregated as the primary data file. To achieve maximum overlap we restricted the analysis to 35–74 years for age-specific estimates of BP and hypertension prevalence, and 35–64 years for age-adjusted results. In the US NHANES whites and blacks were analyzed separately with the appropriate weighting for population size. As previously reported, the prevalence estimates obtained for US blacks from ICSHIB were virtually identical to those from NHANES [6]; to enhance generalizability, however, we used the NHANES data to represent the US black population. Hypertension prevalence and control was age-adjusted by age-averaging the 5-year age groups combining the data for men and women. For comparison of all white vs. all black populations the mean BP's and prevalences were averaged, considering each country as a single unit (i.e., without weighting by population size).

## Results

### Patterns of blood pressure

**Figure 1:**
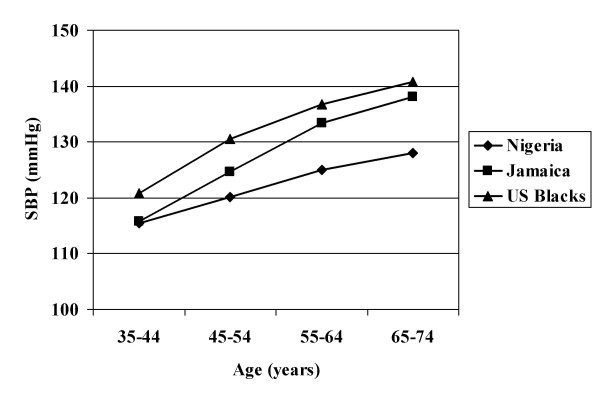
Mean Systolic Blood Pressure, African Descent Populations; By Age Group

**Figure 2:**
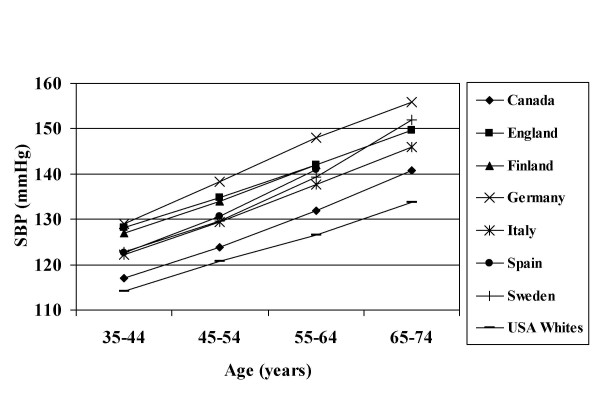
Mean Systolic Blood Pressure, European Descent Populations; By Age Group

**Figure 3:**
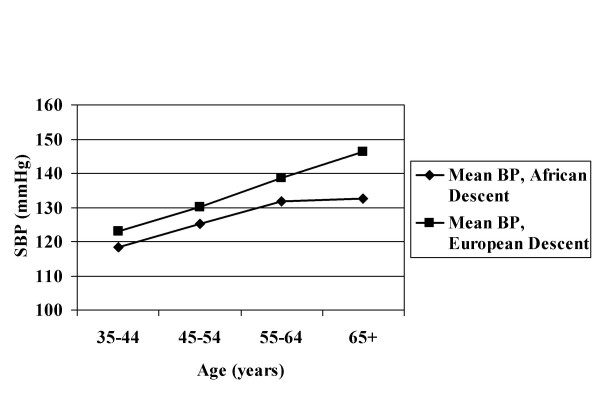
Mean Systolic Blood Pressure, African and European Descent Populations; By Age Group

The age-averaged BPs and BMIs are presented for each survey, by gender, in Table 2. It must be recognized that where treatment is common these data may understate the true values, although this effect is likely to be small when the population is considered as a whole. Trends in BP with age showed considerable heterogeneity within population groups of both continental ancestry (i.e. African and European) (Figures 1, 2). In rural Nigeria, mean BPs were low and rose only modestly with age (Figure 1). Intermediate levels of BP were observed in Jamaica, while the US blacks had higher BPs at all ages. As previously reported, whites in the US and Canada had substantially lower BPs over the entire life span than did the Europeans (Figure 2). For greater clarity, the age-specific patterns are presented for all black and all white groups combined (Figure 3).

**Table 2:** Mean Systolic and Diastolic Blood Pressure and Body Mass Index among Persons 35–74 Years, in African- and European-Origin Populations*

	**Total Sys / Dias**	**Men Sys / Dias**	**Women Sys / Dias**	**BMI, All**
	(mmHg)	(mmHg)	(mmHg)	(kg/m2)
**African-Origin Populations**				
Nigeria	121.5/72.4	122.2/73.0	121.0/71.9	22.9
Jamaica	122.9/71.7	122.5/72.0	123.2/71.5	27.0
US – Black	129.7/78.5	130.3/80.8	129.1/76.3	28.5
**European-Origin Populations**				
US – White	120.9/75.2	123.4/78.2	118.3/72.2	27.3
Canada	128.2/80.8	131.2/83.2	125.1/78.5	26.8
Italy	129.8/83.1	132.4/85.4	127.2/80.7	26.4
Sweden	130.6/80.9	133.0/83.4	128.3/78.4	26.5
England	135.0/77.2	137.3/80.3	132.7/74.2	27.1
Spain	131.4/83.2	132.3/83.9	130.5/82.5	27.4
Finland	134.3/83.8	136.9/86.0	131.6/81.5	27.1
Germany	138.0/86.4	139.5/88.5	137.3/84.3	27.3

* Age-adjusted

### Hypertension prevalence

**Figure 4:**
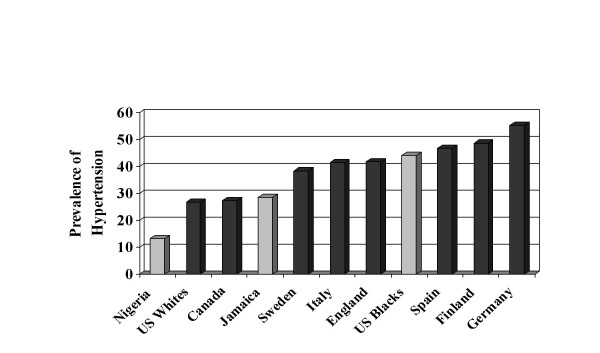
Hypertension Prevalence (140/90 mmHg or Treatment), African and European Descent Populations; Ages 35–64, Age Adjusted

Hypertension prevalence, which accounts for the effect of treatment, follows a similar pattern although the east-west gradient among the African-origin groups is more consistent (Table 3, Figure 4). Among the 14 populations, US blacks fall near the middle in terms of prevalence (mean prevalence = 37%, U.S. blacks = 44%). Among those above the mean, all but one is of European origin. Important differences are apparent in the gender-specific prevalence hypertension in these groups. Among Jamaican women hypertension was substantially more common than among Jamaican men (32% vs. 23%), and relative gender equality existed for US blacks. In Europe, however, the prevalence of hypertension was higher among men in every country (range 5–10%).

### Hypertension prevalence and obesity

The only etiological factor on which standardized information was available was obesity, measured by its proxy BMI. The correlation between average BMI and hypertension prevalence was 0.6 (p < 0.01), all populations combined. Within the black populations the same correlation was observed between mean BMIs and hypertension prevalence (r = 0.6). Among whites, however, the relationship was weaker (r = 0.3). Of course, since obesity will be correlated with many other aspects of lifestyle, it is difficult to infer whether weight gain itself is playing a less important role in determining the variation among white populations. The contrasts noted above in hypertension prevalence by gender are consistent with the relative excess of obesity in women compared to men among Jamaicans and US blacks [2,6].

## Discussion

Comparisons of BP distributions across populations are made difficult by the requirement of comparability of the survey methods. In the last two decades, however, adoption of standardized protocols along with rigorous training have greatly improved the quality of epidemiological studies of hypertension [6,17-19]. A number of countries now conduct recurring national surveys that monitor both secular trends and regional variation within the country [10-12,19,20]. While independent surveys from the same base population had given divergent results in the past, at least two recent single-community studies conducted in the US provided estimates virtually identical to NHANES [21,22]. Although this evidence does not diminish the requirement of careful assessment of survey methodology before making comparisons, it does demonstrate that reliable information can be obtained from independent studies.

The data presented here demonstrate a two-fold variation in prevalence of hypertension in both European- and African-origin populations. The prevalences are similar in blacks in the US and whites in Europe, although important gender differences are apparent. Although not a systematic sample, the populations that are included generally reflect the characteristic social setting in which these groups are found around the world. Summed across all groups, the white populations on average have a substantially higher burden of hypertension. This result can be attributed in large part to the inclusion of several black samples from developing countries where risk factors for hypertension are presently at a lower level. In the only head-to-head comparison within the same survey, US blacks have a prevalence that is 50% higher than among whites. Data from the UK, including the national survey, also demonstrate higher BPs and more hypertension among blacks of Caribbean and African descent [23-27]. On the whole, however, the published literature on racial disparities in hypertension from the UK is less consistent than in the US, where essentially every study has reported higher rates among blacks [28]. Surveys from Cuba, Trinidad and Brazil have also shown a smaller black-white gradient in BP than found in North America [29-31].

Are these findings merely artifactual, reflecting either methodological error or the sampling process? The most unexpected features of the data presented here are the high rates of hypertension in Europe, when contrasted to whites in Canada and the US. These results have been reported in greater detail in an earlier publication [16]. It is beyond the usual standard of statistical significance for the six European surveys to be higher by chance than both of those in North America (p < 0.05). As previously demonstrated, mortality rates for stroke – the most sensitive vital statistics indicator of uncontrolled high BP – are strongly correlated with the prevalence of hypertension among these countries ('r' = 0.8) [16]. Although the data are more limited, hypertension appears to be even more common in Eastern Europe [32-34]. In a comparison of Pol-MONICA with the US-based ARIC study, systolic BPs in Poland were 20 mmHg higher than in the US [3].

The primary purpose of this analysis was to provide descriptive results and very limited information was available on factors that might explain the findings we observed. The gradient among the black populations is consistent with the transition to an industrialized lifestyle and is thereby collinear with most known risk factors [6]. BMI is serving as an effective proxy for this relationship, although its independent contribution cannot be quantified. The explanation of the European-North American contrasts among the white populations is not as apparent. As we have discussed elsewhere, either known risk factors other than obesity are having a larger impact at the population level than usually appreciated, or unknown factors are at work [16]. In either case, further examination of this question seems justified.

Treatment guidelines and practice patterns vary widely among these countries [16-19]. Widespread treatment could, of course, alter the mean BPs in a population, although this effect would be confined to persons over 55 where hypertension is common. The US has the highest rate of treatment, with about 25% of hypertensives controlled, compared to 10% in Europe and less than 1% in Africa (with hypertension defined as 140/90 mmHg)[16]. Any biases that would be introduced into the cross-national comparisons by differential treatment and control are insufficient to alter the primary conclusions, however. The virtual absence of treatment in rural Africa would mean that the natural distribution has essentially been observed unaltered. The effect of treatment in the US or Canada would not be apparent in younger individuals, where contrasts in BPs with Europe and Africa are equally large.

If the North American-European contrasts are occurring in genetically homogeneous populations, large environmental influences must be at work that are not apparent on the surface. A similar process could be taking place across the social environments into which persons of African origin are assorted within societies such as the US and the UK. The debate over inherent susceptibility cannot be resolved with these data since neither the genetic nor the environmental influences can be held constant, allowing a test of the relative influence of the other factor. In fact, the question of inherent susceptibility is probably non-testable under any circumstances [35-37]. While the assumption is often made that contrasting environmental influences between blacks and whites can be adjusted by using proxy measures such as education, that assumption does not hold up under close examination [38]. Perhaps more to the point, however, these data demonstrate that the consistent emphasis given to the genetic elements of the racial contrasts may be a distraction from the more relevant issue of defining and intervening on the preventable causes of hypertension, which are likely to have a similar impact regardless of ethnic and racial background [39]. Once the problem of ethnic/racial contrasts is characterized more closely as a special instance of environmental influences at the population level, it could become more tractable in both the realms of research and practice.

## Competing interests

The author(s) declare that they have no competing interests.

## Authors' contributions

RC, KWM, AL and JB were responsible for study concept, design and supervision. RC, KWM, JB, SG, MJ, AA, TF MK, PP, BS and MT were involved in data acquisition. RC, KWM, JB, MJ, PP and AL were responsible for analysis and interpretation of data. RC and KWM drafted the manuscript. RC, JB, SG, AL, AA, TF, MJ, MK, PP, BS and MT were involved in critical revision of the manuscript for important intellectual content. Statistical expertise was provided by MJ. Administrative, technical and material support was provided by KWM, MK, and BS.

**Table 1:** Characteristics of the Surveys

**Country**	**Survey Yr(s)**	**Population**	**N**	**Male (%)**	**Participation Rate (%)**	**Age Range**	Sampling Method*
Nigeria	1991–93	Local	1931	45	NA	25–74	Multistage, address
Jamaica	1993–95	Local	2573	41	65	25–74	Multistage, address
USA, NHANES Black	1988–94	National	5283	45	82	18–80+	Multistage, population registry
Canada	1986–92	National	23129	49	77.5	18–74	Multistage, medical insurance registries
England	1998	National	11884	45	87.5	16–80+	Multistage, post code address
Finland	1997	National	7064	47	72	25–64	Population registry
Germany	1997–99	National	7047	49	61.4	18–79	Population registry
Italy	1998	National	8233	50	-	35–74	Multistage, population registry
Spain	1990	National	2021	40	73	35–65	Multistage, national registry
Sweden	1999	Regional	1823	49	72	25–74	Population registry
USA NHANES White	1988–94	National	7252	46	82	18–80+	Multistage, population registry

* All stratified sampling methods

## Pre-publication history

The pre-publication history for this paper can be accessed here:


